# Paws for Thought: A Controlled Study Investigating the Benefits of Interacting with a House-Trained Dog on University Students Mood and Anxiety

**DOI:** 10.3390/ani9100846

**Published:** 2019-10-21

**Authors:** Emily L R Thelwell

**Affiliations:** Department of Psychology, University of Warwick, Coventry CV4 7AL, UK; emily.thelwell@warwick.ac.uk

**Keywords:** anxiety, mood, pet therapy, student well-being, dog intervention

## Abstract

**Simple Summary:**

This study investigates whether interacting with a dog would have a positive effect on university students’ mood and anxiety. Students were assigned to either watch videos of a dog or interact directly with a dog. Several measures were collected both before and after their assigned conditions to evaluate their mood and anxiety levels and to assess for possible changes. The results indicated that participants in both conditions experienced a reduction in their anxiety and an improvement in their mood across time, however those who directly interacted with a dog experienced a greater decline in anxiety and improved mood scores.

**Abstract:**

University students have been found to have higher rates of psychological distress than that of the general population, which reportedly rises significantly upon starting university and does not return to pre-university levels throughout their time in university. It is therefore highly important to find ways to improve student health and well-being. One way that may help is by interacting with animals. Therefore, the purpose of this study was to determine whether interacting with a dog would have a positive effect on university students’ mood and anxiety. This study assigned 82 university students to either the experimental condition (dog interaction, *n* = 41) or to the control condition (dog video, *n* = 41). The students completed the Positive and Negative Affect Schedule-Expanded Form (PANAS-X), State-Trait Anxiety Inventory (STAI) and the Pet Attitude Scale before their assigned conditions, to evaluate their mood and anxiety levels and attitudes to animals. The participants again completed the STAI and PANAS-X Form after their condition, to assess for possible changes in anxiety and mood. The findings of the study indicated that all participants, regardless of condition, experienced a reduction in their anxiety and an improvement in their mood across time. However, directly interacting with a dog resulted in greater declines in anxiety and improved mood scores, more so than watching a video. Consequently, it appears there are psychological benefits to be gained by students from interacting with dogs and it is hoped this study will help to inform future best practices in designing student dog interventions.

## 1. Background

University can be a very stressful time for students, especially when they are faced with a new environment as well as the social, academic and emotional challenges that are part of university life. The transition from late adolescence into emerging adulthood is a key developmental period marked by new challenges, changing roles and increased responsibilities [1].

For many students, moving to university is their first time being away from home and they may therefore experience homesickness or have trouble settling while away from friends and family. In particular, first year students are more prone to coping with difficulties when adjusting and learning to handle their new responsibilities. These new challenges may be even more difficult for those individuals who are suffering from psychological issues, such as anxiety and depression. University students have been found to have higher rates of psychological distress than that of the general population [2]. Furthermore, students’ levels of psychological distress have been reported to rise significantly upon initially starting university and do not return to their pre-university levels throughout their time in university [1,3].

Brougham et al. [4] examined the stresses that come from transitioning into university life, the sources of stress and students’ coping strategies. The sources of stress include financial, academic, social, family and daily hassles (e.g., being late). Avoidance, self-punishment and self-help were just some of the coping strategies reported. 

According to the American College Health Association, [5,6]) stress is the most commonly reported barrier to students’ academic success. The American College Health Association’s National Assessment was used to survey American university students with students reporting that within the past year, 58.4% felt overwhelming anxiety, 59% felt very lonely, 65% felt very sad, 37% felt so depressed that they found it difficult to function and 9.8% seriously considered suicide [5,6]. Furthermore, according to the Centre for Collegiate Mental Health 2017 Annual Report [7], 161,014 students at the 147 universities and colleges contributing to the report sought counselling during the academic year, 2016–2017. The three most common psychological issues faced by these students were anxiety (62.2%), depression (49.7%), stress (45.5%). The report also indicated that anxiety and depression are the most common presenting concerns (as assessed by clinicians) and are the only presenting concerns that have demonstrated a clear growth trend over the last four years whilst other concerns appear to be stable. The report also stated that, for the seventh year in a row, the lifetime prevalence rates of threat-to-self characteristics (serious suicidal ideation, non-suicidal self-injury and suicide attempts) increased among students seeking treatment.

In another study, Beiter et al. [8] studied stress, anxiety, and depression among college students. The researchers found that 15% experienced anxiety, 11% struggled with depression and 11% of students reported dealing with stress. The Institute of Public Policy Research [9] analysis suggests that, in the United Kingdom, between the years 2015–2016, 15,395 UK-domiciled first-year students disclosed a mental health condition which is almost five times the number reported in 2006–2007. Furthermore, the report states that between 2007 and 2015, the number of student suicides increased by 79% (from 75 to 134). The number of students who dropped out of university due to experiencing mental health problems had increased by 210% in the years 2014–2015 when compared to 2009–2010 figures. The universities themselves have also reported experiencing significant increases in demand for counselling services over the past five years, with 61% of those universities reporting that the demand has increased by over 25%. 

Given these figures, it is clear that the transition into university can be incredibly stressful, and as a result, this population is particularly vulnerable to developing or exacerbating depressive symptoms [10]. With this in mind, and the impact that these issues may have on students’ academic success, there is a clear need to find ways to reduce stress and improve student health and well-being.

One way that may help is interacting with animals. It is commonly reported in the media that owning a pet can have a positive impact on our physical health and wellbeing, such as increased physical activity, increased social interactions and reduced blood pressure [11]. Studies have also indicated that there are some psychological benefits to be gained from pet ownership. Over the years, research has shown that pet owners, and particularly dog owners, have greater life satisfaction, enhanced self-esteem, reduced levels of loneliness and anxiety, more ambition and more positive moods [12,13,14]. 

Animal assisted therapy (AAT) has been shown to improve mood in children and adults with physical or mental health problems [15,16,17]). There are also a number of studies demonstrating various effects of animals on self-reported anxiety in humans. However, the effects of pets on anxiety are mixed, with some studies finding significant effects and others finding no statistically significant difference [18,19,20,21,22,23,24].

Occasionally reported by the media, are the studies which have shown in some cases a negative effect. Gilbey, McNicholas and Collis [25] administered the UCLA–Loneliness scale to 59 participants living in England who were interested in owning a pet. They then retested the participants 6 months later and found that those individuals who had acquired a pet (35 of the 59) were just as lonely as they had been before getting their pets. Moreover, they were no happier than the participants who had not gotten a pet. Additionally, another study into 117 older adults indicated that those who were considered extremely attached to their dogs tended have higher levels of depression than the participants who did not have as deep an attachment to their dogs [26].

Bao and Schreer [27] sought to explore overall life satisfaction, negative emotions, and positive emotions through two questions and two hypotheses. Firstly, is there a direct correlation with the happiness of the owner whether they owned a pet or not; secondly, whether there was a difference in happiness when comparing dog owners to cat owners [27]. There were 263 participants between the ages of 19–68 who completed numerous questionnaires and the researchers found that there were no significant differences in measured happiness between those with pets and those without. The same analysis was conducted for those people who identified themselves as either a cat person or a dog person and the results showed that there were no differences in positive emotions or overall life satisfaction. However, those individuals who identified as a cat person displayed significantly higher negative emotions than those who identified as a dog person [27]. Therefore, dog visits do not always have a therapeutic effect and there is a need for further research in this area before the benefits can be substantiated. However, whilst previous studies appear to have mixed findings, they do suggest that there may be numerous benefits from AAT programs, including decreased depression and anxiety and it is clear that there is still much to uncover.

Despite the mixed findings of effectiveness, AAT programmes are starting to become increasingly popular on university campuses as they appear to offer an effective option for students struggling with anxiety and stress [28]. The popularity is likely due to the low cost. Most university pet therapy programs are free for students and universities as most of the dog handlers are volunteers [29]). These programmes involve bringing animals and their handlers onto the campus to interact with students. A common format for University AAT programmes involves a large group of students interacting with animals during a single drop-in session [30,31]. The advantage to this format is that a far larger number of students can participate over a shorter period than other format types which require a longer period of time [32] and are more resource-intensive which have been found to have positive effects on students’ mental health and well-being. However, the shorter sessions held in a group format could dilute the immediate as well as the longer lasting benefits of such interventions.

It is only recently that empirical evidence has begun to emerge surrounding the effects of the single drop-in therapy dog session format. Thus far, studies have indicated that a single drop-in group therapy dog session can increase students’ feelings of connection to their campus, temporarily relieve homesickness and stress [33], improve mood and well-being, and decrease anxiety [34]. However, it would appear as though the effects are relatively short-lived as despite an initial recording of effects immediately following the therapy dog session, there were no effects detected two weeks after the sessions took place [33]. Nonetheless there is some qualitative evidence to suggest that the students believe that therapy dog sessions provided them with lasting benefits when asked three months after the sessions [35].

In the United States, 96% of college freshmen were in favour of having a pet therapy programme on campus [36]. It was also recently reported that 62% of surveyed universities in the United States reported having such programmes, many of these exclusively involved dogs [37]. Katcher and Beck [38] revealed that interacting with dogs can reduce physiological indicators of stress, depression, and loneliness. Interacting with dogs has also been shown to encourage fostering social connections with new people [39] as well as reduce anxiety and increase positive affect [40].

A large number of university students suffer from homesickness, whilst for some this is only a minor issue, for others the homesickness can become so severe that they seek out counselling [41]. The usefulness of AAT as a treatment for homesick first-year university students was investigated in a recent study, where a treatment group participated in an 8-week program with trained therapy dogs and their handlers [32]. The students were assigned a 45-min session on the Friday of each week. During these sessions, the student would interact with an assigned dog for 30 min before being allowed to interact with any dog present for the remaining 15 min. A non-treatment control group was informed that they were on a waiting list and never received the treatment. The findings indicated that the intervention was successful in increasing satisfaction with life and decreasing homesickness.

Pets might compliment whatever family support an individual is already receiving or otherwise provide support which is not being provided by a missing family member [42]. One other study found that pets provided social support even for those individuals who are already receiving support from others, and that just thinking about their pets was shown to alleviate the effects of social rejection [43]. Furthermore, Adamle, Riley, and Carlson [36] found that first year students said that their pets provided comfort and support during stressful times. Reduced levels of perceived stress have been observed to be associated with increased happiness and researchers have suggested that finding ways to lower stress may facilitate interventions aimed at reducing depression [44].

The impact pets can have in reducing stress has been well-documented. First year students who were asked to view a presentation about pet therapy before being asked to interact with a therapy dog [36]. The vast majority of the participants said that they missed their pets, that their pets had stayed at home, and that they thought it would be advantageous for therapy dogs to visit campus and help with stress. The researchers also suggested that the students may be helped to form new social connections with others by having access to therapy dogs. Therefore, therapy dogs might also lessen homesickness in students and perhaps enable the students to make new friends and not only reduce stress. This in turn could lead to an overall more enjoyable university experience. Indeed, simply interacting with a dog in pet therapy programmes has also been shown to positively influence college students’ emotional well-being in addition to reducing stress and helping to establish new relationships among the students [45].

Polheber and Matchock [46] investigated various types of social support and its influence on stress reactivity among university students. The participants were randomly assigned to one of three conditions: a novel dog, a friend, or no social support during the experimental procedure. The participants were able to interact with their friend or with the dog before the Trier Social Stress Test (TSST) began—this test contains an arithmetic and speech task. During the Trier stress test, either the friend or dog stayed nearby, or in the case of the no social support group were instructed to sit and relax. The participants’ cortisol levels and heart rates were measured throughout the study. The study found that the participants in the dog condition experienced reduced heart rate and reduced cortisol levels during the TSST in comparison with the other two groups who were not in the presence of a dog. In fact, those who were not in the presence of a dog proved to have higher cortisol levels after the induced stress. The researchers reasoned that this finding was because other humans have the ability to perceive and judge their own friends, whereas dogs can provide a non-judgmental support system for humans [46].

McDonald et al. [47] sought to find a similar result to Polheber and Matchock [46] by examining the effects of a novel dog on University students stress prior to exams. The researchers measured the students’ blood pressure levels both before and after the experiment in two different groups, 15 min prior to a mid-term exam. The first group was instructed that they could do any quiet activity of their choice, whilst the second group was allowed to play with a novel dog. The researchers found that the participants in the group that interacted with a novel dog had significantly lower blood pressure levels after finishing the experiment in comparison to the control group. Interestingly, the control group’s blood pressure was actually found to have increased despite being able to do any quiet activity that they wanted. McDonald et al. [47] determined that exposure to any dog, whether it be trained or untrained, has the potential to reduce blood pressure levels, which can therefore lead to decreased stress in students [47].

Animals have also been shown to have noticeable effects on measurable physiological correlates of stress. Recent research conducted by Somerville et al. [48] examined the effects of physical contact with a dog and a cat on blood pressure and pulse among 62 university students (28 males and 34 females). The participants who held a dog or cat experienced an immediate decrease in diastolic blood pressure. However, this reduction in blood pressure did not occur during the contact with an animal and instead only occurred after the contact had taken place. There were no significant gender differences found, however females did have lower blood pressure than males [48]. Interacting with a dog has also been shown to decrease cortisol levels, and therefore indicate a reduction in stress levels in university students, who themselves did not own a pet. However, this effect was not found for those students who were pet owners [49]. Furthermore, the immunoglobulin IgA, which is another biomarker for stress, was not shown to be affected in either group of participants after interacting with a dog.

Wilson [50] examined the effect of a pet on psychological consequences of stress (i.e., state and trait anxiety levels) of undergraduate students comparing three test conditions (i.e., reading quietly, reading aloud and interacting with a dog). The findings of the study were that reading quietly and interacting with the dog both effected anxiety levels. However, there was more effect seen by reading quietly than by interacting with a dog. There were no significant differences found when examining the interactions among the variables. Whilst a decrease in anxiety level was found after interacting with a pet, pet owners did not report using their pet as a social support significantly more than those who were previously pet owners. Furthermore, whilst the results did indicate that the participants experienced lower physiological and psychological response levels after interacting with a pet, a similar effect was also seen by reading quietly.

In addition to reducing stress, therapy dogs have also been shown in recent research to reduce anxiety levels in university students. Shearer et al. [51] compared the effects that interacting with a therapy dog and mindfulness meditation had on the stress and anxiety levels of students. Whilst interacting with a therapy dog was proven to lower anxiety and stress levels, the mindfulness meditation therapy lowered the students’ anxiety levels more than the sessions with the therapy dog.

However, potentially the biggest disadvantage of owning a pet and therefore of longer-term pet interventions, is the possibility for the animals used to cause distraction from studies, work and other important tasks. Torres et al. [52] found that in their study, the participants were distracted by even just pictures of animals when answering questions on a math exam. Likewise, Foreman et al. [53] in her review of the research saw the potential for these animals to potentially increase unsolicited social attention or cause a distraction from work tasks. However, these studies have both suggested that the occurrence of a distraction could decrease as the novelty of the dog wears off, although more research is needed to show whether this would be the case [52,53]. The other disadvantage to using pets is the impact on the owner or client through the loss and grief experienced after the pet dies. The loss of a pet can be a tremendously emotional event and as a university student, this could cause a distraction from studies and social activities. Furthermore, Eckerd et al. [54] report that many bereaved pet owners experience symptoms of feeling depressed, numbness, crying, feeling guilt, disbelief, or experiencing a sense of loneliness. Given the multitude of different feelings that can be experienced, in addition to the already stressful situations at university, students can be easily distracted from their priorities. This would also be counter intuitive given that the purpose of this type of therapy is to reduce feelings of stress, anxiety and depression.

As outlined, the previous literature on whether pets can have a positive effect on human wellbeing is divided. Even less is known about the effect of owning pets on anxiety and depression in university students and adolescences, as a substantial portion of existing research has focused on the effects of pets on stress in university students or have otherwise focused on mental health in patients and the elderly. However, increasingly high numbers of adolescents are affected by mental health issues and the incidence of mental health issues increases during adolescence, peaking during early adulthood [55].

## 2. The Present Study

Most of the previous research which has investigated the role that dogs may play in the mental health and wellbeing of humans has involved the elderly and children [15,24], with a few studies also looking at the university demographic. However, previous studies that have looked at this demographic have primarily focused on stress as an outcome variable and whilst correlations between stress, anxiety and depression might be drawn, there is little conclusive research surrounding the impact of pets on the latter two within this demographic. This study therefore aims to extend the parameters of this by looking at mood and anxiety.

Furthermore, most of the previous studies have focused on those individuals who are either critically ill or have behavioural disabilities [56,57,58]. Additionally, whilst there are a considerable number of studies that have examined the impact of certified therapy dogs on humans, there is very little research on the benefits of a novel dog on those populations. A novel dog is a typical house-trained dog that is not purposefully trained like therapy dogs to know how to react in response to human emotions, instead working off their own instinct, and are unfamiliar to the participant [47]. This gap in the research was discovered in both studies by Polheber and Matchock [46] and McDonald et al. [47]. Lastly, there are only a few studies which allow participants to have individual sessions, with many studies and AAT sessions operating single drop-in sessions [30,31].

As such, the purpose of the proposed study is to extend research into the effect of pet and human interactions, while also hoping to address some of the limitations. Furthermore, despite the growing body of research on the interaction between humans and animals, the notion that pets have a positive impact on human health and well-being still remains a hypothesis which needs confirmation rather than being an established fact.

This study involved university students interacting with a house-trained 2-year-old female Golden Retriever of which they were unfamiliar. This specific breed of dog was chosen for this study because it is a popular standard breed in the UK and is not considered to be an intimidating or dangerous breed. Retrievers have also been shown to elicit more smiles and verbal responses than other breeds, such as Rottweilers [59]. Additionally, a house-trained dog should provide the most realistic results as possible. Those students who may have a dog at their family homes are more likely to own a typical house-trained dog rather than a therapy dog or will have otherwise come across more house-trained dogs in their lives than therapy dogs.

The question to be addressed is whether interacting with a pet improves mood and reduces anxiety levels. This question is interesting because it would suggest that pets have a positive impact on a person’s physiological state and thus, have potential important health implications for university students through the translation into appropriate interventions concerning pet ownership. At the core, this research aims to ask, “Can students’ mood and anxiety be enhanced from spending time with a pet dog brought to campus?”.

To examine whether interaction with a dog might impact college students’ mood and anxiety levels, a quasi-experimental two group pre/post design study was conducted. It was hypothesised that there would be a significant positive effect on those university students’ mood and anxiety levels that had experienced physical interaction with a dog, compared to the control group that watched a video montage of Golden Retriever clips.

On an exploratory basis, possible gender differences were additionally examined. It has been shown that female students benefit more from therapy dog sessions than male students [35], therefore suggesting the need for research into gender differences.

## 3. Methods

### 3.1. Ethical Considerations

It is important to consider ethics when dealing with studies that involve humans and animals. It is important to keep in mind what the dog is being subjected to and if the activities are fair, as well as if it is a fair environment for the dog. Ethical considerations when conducting research with human subjects are equally important. When testing for aversive or stressful situations or asking questions that could be potentially distressing, it is essential to ensure it is not damaging or harmful to the person [52].

Therefore, ethical approval was sought from the University of Warwick in March 2018 and was approved in May 2018. Additionally, approval was sought and granted by the Animal Welfare and Ethical Review Body (AWERB.48/17-18) as well as the University’s Health and Safety department. Third party insurance was also obtained for the dog participating in the study and the documentation was provided to the insurance office.

For the duration of the study, it was always ensured that a first aider was present during the testing times. The room cleaning team were always made aware that the room needed to be cleaned after every testing session and on one occasion being asked to return to ensure a thorough removal of hair. The dog was checked not to have a history of biting people and that this was not considered a dangerous breed.

The dog in this experiment was granted the ethical approval to spend 10 min with each of 3 participants before receiving a minimum of a 30 min break where it had access to water and was allowed to urinate or defecate as required (totally a time period of 90 min). These 90-min sittings were to be repeated no more than three times per day. The dog would perform these for a maximum of 4 days in a row before being given a minimum 2 days break.

The dog’s usual daily routine was maintained during the testing periods. The dog was constantly monitored for signs of distress by its owner both during and after the sessions and did not display any concerns. If the dog had displayed signs of distress, the study would have stopped until the dog was well enough to resume or otherwise, a replacement would have been found.

### 3.2. Participants

The participants in this study were 82 students who were attending the University of Warwick. Of those 82 students, 42 identified themselves as male, 40 as female, 0 as other (please describe if you wish) and 0 chose the option prefer not to say. Their age range was from 18 to 23 years old. The students were studying a range of subjects and were a mix of first year (*n* = 44), second year (*n* = 12), third year (*n* = 10) and post graduate (*n* = 16). The students were recruited through a posting on the participant recruitment website Sona and did not receive any form of reward for participating in the study. The participants were screened on the sign-up page as well as via a follow up email from the researcher for allergies towards dogs.

### 3.3. Measures

*Demographic survey.* The participants were requested to provide some demographic information that consisted of nine questions including age, gender, ethnicity, what year of study they were in, if they have a pet dog, how frequently they interact with a dog and if they consider themselves as a dog person or a cat person.

*Pet Attitude Scale (PAS).* This measure included 18 statements rated on a 7-point Likert scale from 1 (strongly disagree) to 7 (strongly agree) [60]. The participants were asked to rate certain statements such as “I love pets” or “I spend time every day playing with my pet (or I would if I had one).” The current study had a Cronbach’s alpha of 0.702.

*State-Trait Anxiety Inventory (STAI).* This measure included six statements that were rated on a 1 (not at all) to 4 (very much) Likert Scale [61]. The participants reported how they felt in the moment, involving statements such as “I feel tense” or “I feel content”. The current study had a Cronbach’s alpha of 0.816.

*Positive and Negative Affect Schedule-Expanded Form (PANAS-X).* This measure involved 60 words and phrases that described different feelings and emotions on a 1 (very slightly or not at all) to 5 (extremely) Likert Scale [62]. The participants indicated how much they felt certain emotions, such as how happy, upset or nervous they felt in the moment. Negative affect had a Cronbach’s alpha of 0.902, Sadness scores had a Cronbach’s alpha of 0.868. Positive affect had a Cronbach’s alpha of 0.929 and joviality scores had a Cronbach’s alpha of 0.920.

### 3.4. Procedure

The participants were assigned to one of two groups, the experimental or control. The assignment was based upon which timeslot the participants chose, since each of the two conditions were held on different days unbeknownst to the participants. Seven days were dedicated to the participants coming in spread out over the course of a three-week time period. The first week of the experiment was dedicated to the experimental group and the second week was dedicated to the control group. During the third week, the allocation of the group was dependent on the dogs previously allocated breaks and daily routine. Those participants who had selected a timeslot which coincided with the dog’s arranged break would participate in the control variable. When all participants arrived for their selected timeslot, they were asked to read an information sheet, with the appropriate adjustments to reflect the activity in which these participants were going to engage and were given their own unique identification code. The participants were informed of their right to withdraw at any given point in the study. They would then be asked to fill out the consent form, demographic questionnaire, PAS, STAI and PANAS-X before the experiment was conducted.

**Experimental group:** Those participants in the experimental group (*n* = 41) played with the dog for ten minutes. The time was recorded on an iPhone stop watch to ensure the time for all participants was consistent. Before introducing the participants to the dog, the participants were asked to verbally confirm that they were happy to be in a room with and to interact with a dog. The participants were also asked to verbally confirm once again that they had no allergies to dogs. Only upon this confirmation were the participants allowed to enter the room. The dog that participated in the experiment was a 2-year-old female Golden Retriever. The dog was consistently energetic and eager to meet each new participant and play. The participants were informed they could freely play with the dog, including sitting with her, petting her, throwing toys which squeaked for her, talking to her, and cuddling her. Some participants would be more enthusiastic in their free play than others and would more actively engage with her, whilst others preferred to remain in one place and fuss her quietly.

**Control group:** Those in the control group (*n* = 41) watched a ten-minute-long video montage which was created by the researcher. This video featured multiple amusing or cute clips of Golden Retrievers, the majority of which were of the retriever used in the study, the others were acquired from YouTube. The video was always played on a HP Spectre x360 laptop and the screen brightness was the same for each participant.

After the participants in each condition had finished their dog-related activity, they were requested to complete the post-test surveys (PANAS-X and STAI) to determine if there were any changes to their mood and anxiety scores when compared to their pre-test survey scores. Upon completion of the surveys, a debriefing statement was issued for the participants to read and take with them. Each session lasted approximately forty minutes, including the time to complete the established measures.

## 4. Results

The data was analysed to investigate the study’s hypothesis that there would be a significant positive effect on those university students’ mood and anxiety levels in those students that had experienced physical interaction with a dog, compared to the control group that watched a video montage of Golden Retriever clips. Both groups’ pet attitude analyses scores were firstly examined to ensure that the participants’ attitudes toward pets did not differ between groups, as well as age, gender and whether they consider themselves to be a cat or dog person, or neither. Following this, the mood and anxiety scores were explored as a function of group and time (before exposure to dogs and after).

### 4.1. Preliminary Analyses

**Age:** An independent *t*-test was run to explore whether or not the participants’ ages were significantly different for the experimental condition compared with the control condition. No statistically significant difference was found between conditions for the participants’ ages (experimental group M = 19.66; control group M = 19.76), *t*(80) = −0.366, *p* = 0.716. Therefore, each of the conditions contained participants of a similar age.

**Gender:** A chi-square test was run to explore whether or not the participants’ genders were significantly different for the experimental condition compared with the control condition. No statistically significant difference was found between conditions for the participants’ genders χ(1) = 0.195, *p* = 0.659. The control group consisted of 19 females and 22 males, the experimental group consisted of 21 females and 20 males. Therefore, each of the conditions contained an overall equal split of genders.

**Dog or Cat person:** A chi-square test was run to explore whether or not the split between dog and cat people was significantly different for the experimental condition compared with the control condition. No statistically significant difference was found between the conditions for whether the participants considered themselves a cat or a dog person χ(2) = 0.157, *p* = 0.925. There was a total of 4 participants who considered themselves a cat person (experimental = 2, control = 2), 71 dog people (experimental = 35, control = 36) and 7 participants who considered themselves neither (experimental = 4, control = 3). Therefore, each of the conditions contained an overall equal split of cat and dog people.

**Pet attitude scores:** An independent *t*-test was run to explore whether or not the participants’ pet attitude scores were significantly different for the experimental condition compared with the control condition. No statistically significant difference was found between the conditions for the pet attitude scores (experimental group M= 57.46; control group M = 55.56), t(80) = 5.48, *p* = 0.135. Therefore, each group of participants had similar attitudes towards pets overall.

### 4.2. Main Analyses

#### 4.2.1. STAI Anxiety Scores

**Figure 1:**
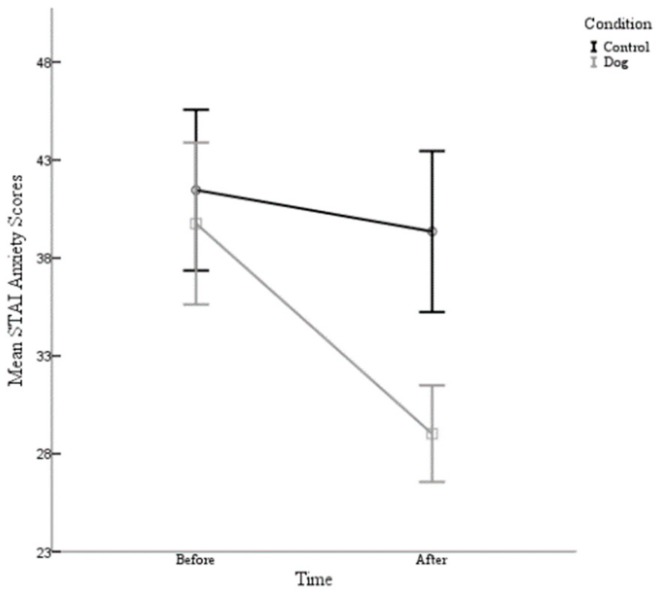
The mean State-Trait Anxiety Inventory (STAI) anxiety scores are plotted for the participants in the control condition and the participants in the experimental (dog) condition before their interaction and after their interaction with 95%-confidence-interval error bars.

A 2 condition (control and dog) × 2 time (Time 1 and Time 2) repeated measures ANOVA conducted on anxiety scores was also performed. The post hoc comparisons using the Bonferroni correction indicated that there was a main effect for condition (F (1,80) =6.01, *p* =0.016). Specifically, the participants in the experimental condition (Before M =39.76, After M=29.02) reported having lower anxiety scores than the control group (Before M = 41.46, After M = 39.35). There was also a main effect for time (F (1,80) = 43.14, *p* = 0.000). Particularly, the participants reported having lower anxiety scores in the post survey (time 2) (M = 34.19) than at Time 1 (M = 40.60), see Table 1. Furthermore, there was a significant interaction between condition and time (F (1,80) = 19.42, *p* =.000) *d* = 0.985. As can be seen in Figure 1, the participants in this study experienced a decrease in their anxiety scores following their exposure to dogs, whether they directly interacted with a dog or simply observed dogs on a video. However, this was significantly greater in the experimental condition than the control.

#### 4.2.2. PANAS-X Negative Affect Scores

A 2 condition (control and dog) × 2 time (Time 1 and Time 2) ANOVA conducted on the general negative effect was performed. The post hoc comparisons using the Bonferroni correction indicated that there was no main effect for condition found (F (1,80) = 0.078, *p* = 0.780). Specifically, the participants in the dog condition (Before M = 15.12, After M=12.15) did not report having a lower general negative affect than the control group (Before M = 14.24, After M = 13.54). However, there was a main effect for time (F (1,80) = 27.48, *p* = 0.000). Particularly, the participants reported having a lower general negative affect in the post survey (M = 12.84) than at the pre survey (M = 14.68), see Table 2. There was also a significant interaction between condition and time (F (1,80) = 10.42, *p* = 0.002) *d* = 0.722.

**Figure 2:**
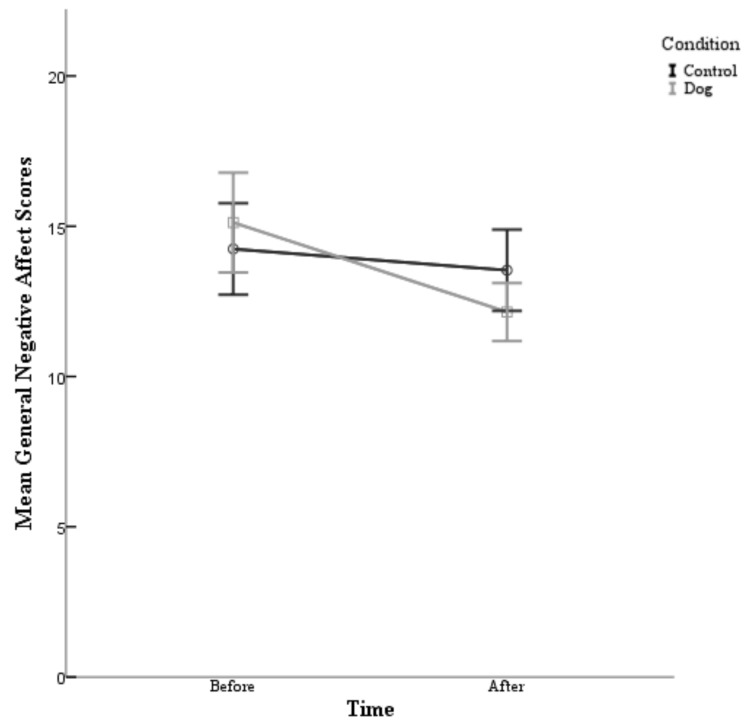
The mean general negative affect scores are plotted for the participants in the control condition and the participants in the experimental (dog) condition before their interaction and after their interaction with 95%-confidence-interval error bars.

As can be seen in Figure 2, the participants in this study experienced a decrease in their general negative scores following their exposure to dogs, whether they directly interacted with a dog or simply observed dogs on a video.

#### 4.2.3. PANAS-X Sadness Scores

**Figure 3:**
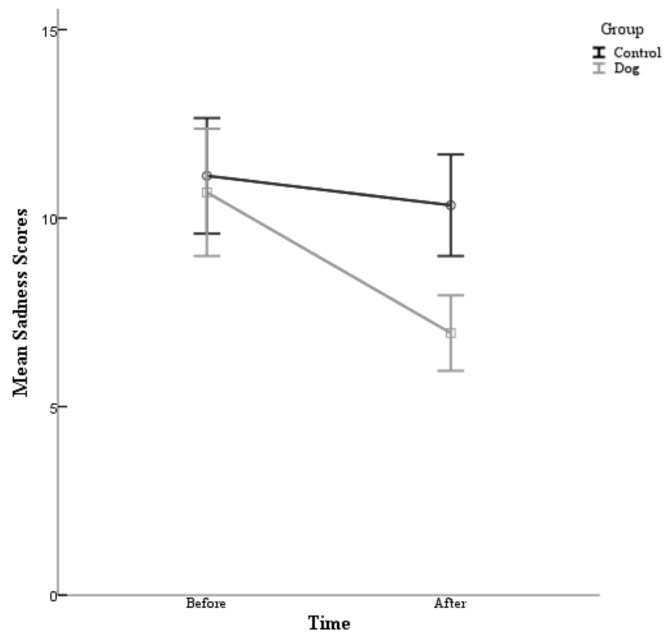
The mean sadness scores are plotted for the participants in the control condition and the participants in the experimental (dog) condition before their interaction and after their interaction with 95%-confidence-interval error bars.

A 2 condition (control and dog) × 2 time (Time 1 and Time 2) repeated-measures ANOVA conducted on sadness scores was also performed. A main effect for condition was found (F (1,80) = 4.24, *p* = 0.043). Specifically, the participants in the experimental condition (Before M = 10.68, After M = 6.95) reported lower sadness scores than the control group (Before M = 11.12, After M = 10.34). There was also a main effect for time (F (1,80) = 43.30, *p* = 0.000). Particularly, the participants reported having lower sadness scores in the post survey (M =8.65) than at the pre survey (M = 10.90), see Table 3. Furthermore, there was a significant interaction between condition and time (F (1,80) = 18.53, *p* = 0.000) *d* = 0.963. As can be seen in Figure 3, the participants in this study experienced a decrease in their sadness scores following their exposure to dogs, whether they directly interacted with a dog or simply observed dogs on a video. However, this was significantly greater in the experimental condition than the control.

#### 4.2.4. PANAS-X General Positive Affect Scores

A 2 condition (control and dog) × 2 time (Time 1 and Time 2) repeated-measures ANOVA conducted on general positive affect was performed. The post hoc comparisons using the Bonferroni correction indicated that there was no main effect for condition found (F (1,80) = 16.76, *p* = 0.725). Specifically, the participants in the experimental condition (Before M = 26.71, After M = 30.37) did not report having a higher general positive affect than the control group (Before M = 27.98, After M = 28.07). There was however a main effect for time (F (1,80) = 16.76, *p* = 0.000). The participants reported having a higher general positive affect in the post survey (M = 29.22) than before the survey (M = 27.34), see Table 4. There was also a significant interaction between condition and time (F (1,80) = 15.06, *p* = 0.000) *d* = 0.868. Therefore, the participants in the experimental condition and the control condition did not experience a significant increase in their positive affect scores following their interactions.

**Figure 4:**
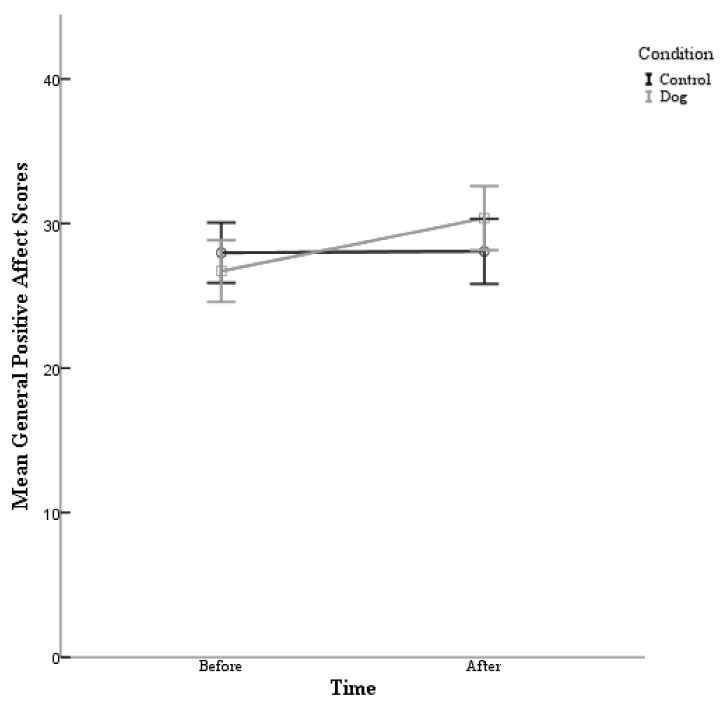
The mean for general positive affect is plotted for the participants in the control condition and the participants in the experimental (dog) condition before their interaction and after their interaction with 95%-confidence-interval error bars.

As can be seen in Figure 4, the participants in this study experienced an increase in the general positive affect scores following their exposure to dogs, whether they directly interacted with a dog or simply observed dogs on a video.

#### 4.2.5. PANAS-X Joviality Scores

A 2 condition (control and dog) × 2 time (Time 1 and Time 2) repeated-measures ANOVA conducted on joviality scores was also performed. The post hoc comparisons using the Bonferroni correction indicated that there was a main effect for condition (F (1,80) = 60.23, *p* = 0.000). Specifically, the participants in the experimental condition (Before M = 23.15, After M = 29.29) reported having higher joviality scores than the control group (Before M = 23.22, After M = 24.05). There was also a main effect for time (F (1,80) = 34.99, *p* = 0.000). Particularly, the participants reported having higher joviality scores in the post survey (M = 26.67) than at the pre survey (M = 23.18), see Table 5. Furthermore, there was a significant interaction between condition and time (F (1,80) = 4.16, *p* = 0.045) *d* = 0.456.

**Figure 5:**
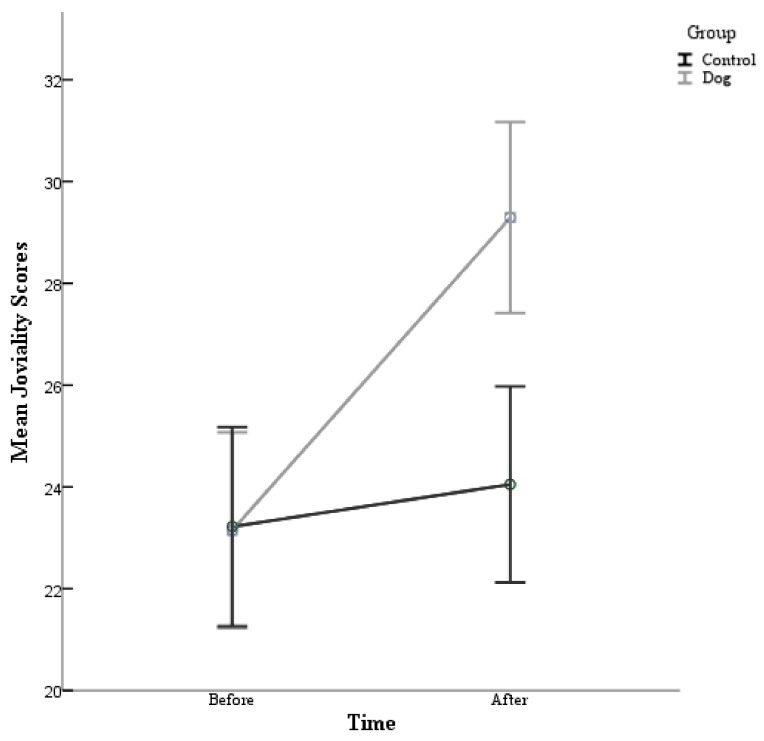
The mean joviality scores are plotted for the participants in the control condition and the participants in the experimental (dog) condition before their interaction and after their interaction with 95%-confidence-interval error bars.

As can be seen in Figure 5, the participants in this study experienced an increase in their joviality scores following their exposure to dogs, whether they directly interacted with a dog or simply observed dogs on a video. However, this was significantly greater in the experimental condition than the control.

### 4.3. Exploring Gender

#### 4.3.1. STAI Anxiety

Anxiety scores: A 2 gender (male and female) × 2 condition (control and dog) X 2 time (Time 1 and Time 2) repeated-measures ANOVA conducted on anxiety scores was also performed. There was no significant effect found for gender (F (1,78) = 0.004, *p* = 0.951), meaning there were no significant differences between the two genders. Specifically, the female participants’ scores (Before M = 39.92, After M = 41.05) were not significantly different from the male participants’ scores (Before M = 41.27, After M = 33.97), see Table 6. There was also no interaction found for time and gender (F (1,78) = 1.28, *p* = 0.261) nor was an interaction found between gender and group (F (1,78) = 0.893, *p* = 0.348). There also was no significant interaction between time, gender and group (F (1,78) = 0.016, *p* = 0.900).

#### 4.3.2. Negative Affect Scores

A 2 gender (male and female) × 2 condition (control and dog) × 2 time (Time 1 and Time 2) repeated-measures ANOVA conducted on general negative scores was also performed. There was no significant effect found for gender (F (1,78) = 0.078, *p* = 0.780), meaning there were no significant differences between the two genders. Specifically, the female participants’ scores (Before M = 14.93, After M = 12.88) were not significantly different from the male participants’ scores (Before M = 14.45, After M = 12.81), see Table 7. There was also no interaction found for time and gender (F (1,78) = 0.535, *p* = 0.467) nor was an interaction found between time, gender and group (F (1,78) = 0.445, *p* = 0.507). There was however a significant interaction between group and gender (F (1,78) = 0.078, *p* = 0.041). Specifically, the males in the control condition had a much lower negative affect scoring before the intervention (M = 12.90) than those males placed in the dog condition (M = 15.86), but those in the dog condition had the greater decline in their negative affect scores after the intervention took place (M = 12.91) than the control (M = 12.70).

#### 4.3.3. Sadness Scores

A 2 gender (male and female) × 2 condition (control and dog) × 2 time (Time 1 and Time 2) repeated-measures ANOVA conducted on sadness scores was performed. There was no significant effect found for gender (F (1,78) = 0.694, *p* = 0.407), meaning there were no significant differences between the two genders. Specifically, the female participants’ scores (Before M = 10.50, After M = 8.35) were not significantly different from the male participants’ scores (Before M = 11.29, After M = 8.93), see Table 8. There was also no interaction found for time and gender (F (1,78) = 0.008, *p* = 0.927) nor was an interaction found between group and gender (F (1,78) = 0.552, *p* = 0.460. There was also no a statistically significant three-way interaction between time, gender and group (F (1,78) = 0.211, *p* = 0.648).

#### 4.3.4. Positive Affect Scores

A 2 gender (male and female) × 2 condition (control and dog) × 2 time (Time 1 and Time 2) repeated-measures ANOVA conducted on positive affect scores was performed. There was no significant effect found for gender (F (1,78) = 0.498, *p* = 0.483), meaning there were no significant differences between the two genders. Specifically, the female participants’ scores (Before M = 27.95, After M = 29.63) were not significantly different from the male participants’ scores (Before M = 26.76, After M = 28.83), see Table 9. There was also no interaction found for time and gender (F (1,78) = 0.058, *p* = 0.811) nor was an interaction found between group and gender (F (1,78) = 1.81, *p* = 0.182. There was also no significant interaction found between time, gender and group (F (1,78) = 0.055, *p* = 0.815).

#### 4.3.5. Joviality Scores

A 2 gender (male and female) × 2 condition (control and dog) × 2 time (Time 1 and Time 2) repeated-measures ANOVA conducted on joviality scores was performed. There was no significant effect found for gender (F (1,78) = 0.737, *p* = 0.393) meaning there were no significant differences between the two genders. Specifically, the female participants scores (Before M = 23.75, After M = 27.08) were not significantly different from the male participants scores (Before M = 22.64, After M = 26.29), see Table 10. There was also no significant interaction found for time and gender (F (1,78) = 0.004, *p* = 0.949) as well as for group and gender (F (1,78) = 2.38, *p* = 0.127). Nor was there a statistically significant three-way interaction between time, gender and group (F (1,78) = 0.055, *p* = 0.815).

## 5. Discussion

The purpose of the present study was to determine whether there would be a positive effect on university students’ mood and anxiety scores after interacting with a dog. Consistent with past research, the results of this study do provide evidence that interacting with a dog does have a positive effect on the students’ emotional well-being. This study found considerable increases in happiness and reductions in both anxiety and sadness scores immediately after the students interacted with a dog.

The findings of this study are in line with previous research indicating there are short-term psychological benefits of interacting with a dog [33,34,38,40,47]. However, this study fills a research gap as unlike much previous research on this topic which uses a typical single drop-in group therapy dog session [30,31], this study was designed to allow the students to interact with the dog on an individual basis. As past research has primarily focused on children and the elderly or otherwise the impact on stress in students, there has been limited research conducted on the university population specifically with regards to the role of dogs influencing anxiety and depression. Therefore, this study aimed to fill this research gap.

### 5.1. Anxiety

It was hypothesised that there would be a significant positive effect on those university students’ anxiety levels that had experienced physical interaction with a dog, compared to the control group who watched a video montage of Golden Retriever clips. In the present study, this hypothesis was supported because a main effect for condition was found and the participants in the dog condition reported having lower anxiety scores than in the control group who watched a video. Thus, it appears that the treatment did have a positive effect on the anxiety levels of the university students. This result supports the previous research findings of Cole et al. [21], in which it was found that hospital patients experienced the greatest decrease from baseline in state anxiety scores after interacting with a dog when compared with the other groups. A main effect of time was also found for anxiety, with participants reporting lower levels of anxiety in the post survey for both conditions. As there was also a significant interaction between condition and time, it can be concluded that the participants in this study experienced a decrease in their anxiety scores following their exposure to dogs, whether they directly interacted with a dog or simply observed dogs on a video. However, given the main effect of condition, this was significantly greater in the experimental condition than the control.

### 5.2. Negative Mood

Negative mood was looked at in two ways. Firstly, general negative affect was examined which totalled the scoring for responses of feeling afraid, scared, nervous, jittery, guilty, ashamed, irritable, hostile, upset, distressed. Secondly, sadness was examined which included responses of feeling sad, blue, downhearted, alone, lonely. It was hypothesised that that there would be a significant positive effect on those university students’ mood levels that had experienced physical interaction with a dog, compared to the control group who watched a video montage of Golden Retriever clips.

**Negative affect:** With regards to negative affect scores, there was no main effect for condition found. However, a significant interaction between condition and time was found. Specifically, the participants in both the dog condition and control condition did experience a significant decrease in general negative affect following their interactions. The participants also reported having lower general negative affect in the post survey than at the point of the pre-survey, regardless of group. This would appear to be in line with the likes of Wilson [50] where there was shown to be a positive impact found on participants from a dog interaction condition, however, a parallel effect was also seen by reading quietly.

**Sadness scores:** With regards to sadness scores, the hypothesis was supported as a main effect for condition was found and the participants in the experimental condition reported having lower sadness scores than the control group. Thus, it appears that the treatment did have a positive effect on the sadness scores of the university students. There was also a main effect for time and a significant interaction between condition and time. Therefore, the participants in this study experienced a decrease in their sadness scores following their exposure to dogs, whether they directly interacted with a dog or simply observed dogs on a video. However, this was significantly greater in the experimental condition than the control. This result supports the previous research findings of Orlandi et al. [14] who compared two groups of patients receiving chemotherapy, with one group serving as a control and the other group assigned to receiving a visit by a therapy dog during the chemotherapy session. The results of their study indicated that depression levels only showed improvement in the dog visit group and not in the control group.

### 5.3. Positive Mood

Positive mood was also looked at in two ways. Firstly, general positive affect was examined which totalled the scoring for responses of feeling active, alert, attentive, enthusiastic, excited, inspired, interested, proud, strong, determined. Secondly, joviality was examined which included responses of feeling cheerful, happy, joyful, delighted, enthusiastic, excited, lively, energetic. It was hypothesised that that there would be a significant positive effect on those university students’ mood levels that had experienced physical interaction with a dog, compared to the control group.

**Positive affect:** With regards to positive affect scores, there was no main effect for condition, however there was a main effect of time as well as a significant interaction between condition and time. Therefore, the participants in the experimental condition and control condition experienced a significant increase in their positive affect scores, regardless of which interaction group they were placed. This would appear to support research conducted by Bao and Schreer [27] who explored overall life satisfaction, negative emotions, and positive emotions. The researchers found that there were no significant differences in measured happiness or overall life satisfaction between those with pets and those without.

**Joviality scores:** However, upon analysis of the joviality scores, the hypothesis was supported as a main effect for condition was found and the participants in the experimental condition reported having higher joviality scores than the control group. Thus, it appears that the treatment did have a positive effect on the joviality scores of the university students. There was also a main effect for time and a significant interaction between condition and time. Therefore, the participants in this study experienced an increase in their joviality scores following their exposure to dogs, whether they directly interacted with a dog or simply observed dogs on a video. However, this was shown to be significantly greater in the experimental condition than the control. This result also supports the previous research findings of Orlandi et al. [14] in addition to El-Alayli et al. [12], Nathans-Barel et al. [16] and Kaminski et al. [15] who reported significantly greater positive moods after participants were involved with dog therapy in comparison with control groups.

### 5.4. Gender Differences

There were no statistically significant results found between genders for any of the variables in this study, apart from the variable of negative affect. There was a statistically significant effect found between group and gender within this variable with males placed in the dog condition reporting a higher initial negative affect score before the intervention took place and experiencing a greater drop after the intervention. This contradicts previous research which suggests that female students benefit more from therapy dog interventions than males [35]. However, overall there were minimal differences between genders.

### 5.5. Strengths and Limitations

One strength of this study was its participant size. There were 82 students who participated in this study, ranging between all study years. Whilst the majority of students who took part were white (*n* = 48), this study also included multiple other ethnicities including Chinese (*n* = 5), Asian or Asian British—Indian (*n* = 13), Asian or Asian British—Pakistani (*n* = 2), other Asian (*n* = 3), other (n = 7), Black British (n = 3) and other White background (*n* = 1). However, as the sample of students consisted solely of students studying at the University of Warwick, the findings may not be generalised to other universities. That being said, as mentioned above, there was a diverse range of participants in the present study which may help the generalisability.

However, as with all research, there are several limitations with the present study. Firstly, one of the biggest limitations with this study was that those individuals who took part were largely those who considered themselves as a dog person (*n* = 71) and as such may have experienced the greatest benefit from spending time in the dog interaction condition. People who do not like dogs did not appear to be as likely to sign up to these types of studies and so therefore, the effect sizes may be overestimated. Furthermore, the large number of participants who consider themselves a dog person suggests that there could be a self-selection bias. Perhaps, one of the biggest limitations to most of these studies is that great portions of the subjects were already shown to either have an interest in or a general like for dogs. Numerous previous studies have also used a pet attitude scale to determine if the participants either have a strong liking for dogs or otherwise, a dislike of dogs prior to beginning the study [46]. This can be a strength as there is a better chance that the study will be more successful if the participants in the study are already proven to be fond of spending time with a dog. Regrettably, having a larger number of participants who consider themselves as dog people also decreases the generalisability of the study, given that it is only applicable to the part of the population who either like dogs or are not afraid of them. Furthermore, reviews of animal-human relationship studies have also found that typically, these studies rarely display the effects of other pets on humans and generally only account for mainly dogs, and less frequently cats [27]. Secondly, a potential limitation of this study was that it was a controlled and not a completely random study. Whilst the participants selected which times and days they were available, some people with particular traits may have only been able to make it in the first week while others in the second week and therefore, this might add bias to the current study by not having randomisation. Finally, the demographic data collected in this study did not include sexual orientation, social factors or disability which are known to affect mental health [63,64,65,66].

### 5.6. Future Research

It should be noted that not all people like dogs. Some people may have a phobia of dogs, may be allergic to dogs, or may simply just not want to interact with a dog [67]. Therefore, future research could include more of those individuals who rate themselves as “neither” and “cat people” to examine the differences in the effects, as those who do not like dogs may find the experience unpleasant and may instead experience an increase in anxiety levels and negative mood. Additionally, future research should also include other house-trained pet types in order to examine the effects on parts of the population who prefer other animals over dogs.

The researchers also believe that it may have been more beneficial for the study and sessions to take place during particularly stressful periods of the study year, for instance it would have been preferable for the study to take place at the start of the year when university life is new, particularly for undergraduates, and students are transitioning to university life as this is considered to be a stressful time, and as a result this population is particularly vulnerable to developing or exacerbating depressive symptoms [10]. Other stressful periods of time could be during exam periods, or even for dogs to be present while students complete assignments. This is something to consider with regards to future research. However, this study took place just after the end of year exams while students were waiting for their results which might also considered to be a stressful point of their year. The directions for future research could include the research taking place from the beginning of the year through to the end at regular intervals to compare times of elevated stress, especially for first-year students, and should go beyond the temporary relief offered by one-off sessions and incorporate longitudinal measures into the design to examine if there is a long-term benefit of interacting with a dog for students. Do the effects dissipate over time? How short-lived are the effects? Future studies could incorporate several measurement points. Moreover, is there an association between university students’ productivity throughout the year and interacting with a pet regularly? Whilst there is research to suggest an association between reduced stress, anxiety and depression and dog therapy sessions, it is not yet known whether this reduction has an impact on university students’ productivity and academic success. Might the quality and amount of work accomplished be higher due to the psychological benefits provided by owning a pet, or could they in fact be lower as a result of added responsibility and distractions? Generally, with all populations, there are limited longitudinal studies that have examined the impact of pets, therefore to investigate what exactly are the lasting impacts that dogs have on humans, it would be very beneficial to utilize such designs [53].

It is also important to consider cultural differences with regards to using pets in therapy sessions, as not all cultures feel the same way about domestic pets, such as dogs. Not everyone shares the same perception or love for animals or see them as a positive influence on their life [53]. It is therefore vital to bear in mind that the concept of animals as a therapeutic intervention is not universally accepted as some people might have cultural or religious reasons for why they do not want to interact with animals [68]. This could open avenues for future research to ensure that animal interventions are culturally suitable and beneficial, as not much is known about how cultural or religious views impact therapeutic outcomes in animal interventions [69]. Though researchers and animal volunteers should mitigate such risks by asking beforehand whether the potential individual is happy to interact with the animal in question. Equally, it is important to bear in mind that these interventions involve living, breathing animals and there are risks to the participating therapy dogs themselves [67]. Therefore, it is mandatory that those in charge of organising and running such interventions should ensure the therapy dogs’ health, safety, welfare, and that its daily routine is maintained.

With this is mind and given that the number of animal interaction studies are increasing, future research should consider investigating the impact such interventions on the therapy dogs themselves as at present, the research in this area is lacking [70]). There are only a few animal intervention studies that incorporated measures to investigate how participating therapy animals are affected by these studies [71]. Therefore, in order to be considered humane and effective methods of treatment, animal interventions should be beneficial to both the participating therapy animals and the potential clients [70] and researchers should investigate the effects of these interventions on both parties given the current lack of clarity.

Future research should also consider exploring which of the specific aspects of university dog sessions lead to better lasting positive effects, for instance, explore what the optimal number of sessions is for long-term benefits or what the optimal length of time is for students to remain engaged with the session. In addition to using self-report measures, future research could also include the use of physiological measures, such as heart rate, galvanic skin response, cortisol levels and blood pressure to assess changes. Given that students’ levels of psychological distress have been reported to rise significantly upon initially starting university and do not return to their pre-university levels throughout their time in university [1,3], it is of high importance that students have accessible interventions to reduce anxiety and depression.

## 6. Conclusions

To the best of the researcher’s knowledge, this present study helps to fill a research gap and provides further support that allowing students to interact with dogs on campus has positive psychological benefits to students. Whilst no significant results were found between the groups for negative or positive affect, the researchers argue that the items that comprised the sadness and joviality scales were more relevant to mood and feelings, while affect is more the experience of their emotions. This would however appear to be in line with previous research that has suggested that animals do not necessarily even need to be present and simply images alone of pets can improve psychological wellbeing [52]. This study found that there were positive effects on anxiety, joviality and sadness in the case of the control variable as with the experimental, though the benefits of the experimental condition were significantly greater, and there were positive effects on general positive affect and general negative affect regardless of condition. Ultimately, the use of dogs whether they have been trained specifically for therapy or are general house-trained dogs, or even just the use of videos, can be a highly beneficial tool for universities to implement to help reduce depression and anxiety in students. The positive benefits associated with students being allowed to interact with dogs in university appears to be a suitable, relatively low-cost and effective way to enhance and sustain student psychological well-being.

To conclude, the results of this study indicate that interacting with a pet dog considerably reduces anxiety and feelings of sadness and improves happiness scores. There are several limitations with the current study which leave a gap for future studies to fill. Further controlled studies are required for confirmation and to accurately define the population who will receive the greatest benefit from such treatments, as well as the optimum duration. It is hoped that this study will help to inform future best practices in designing student dog interventions, which will in turn, facilitate improved psychological well-being in university students.

## Figures and Tables

**Table 1:** Descriptive Statistics: Anxiety.

	Condition	Mean	Std. Deviation	N
Before	Control	41.46	13.02	41
	Dog	39.76	13.11	41
	Total	40.61	13.01	82
After	Control	39.35	13.02	41
	Dog	29.02	7.83	41
	Total	34.19	11.87	82

**Table 2:** Descriptive Statistics: Negative Affect.

	Group	Mean	Std. Deviation	N
Before	Control	14.24	4.821	41
	Dog	15.12	5.269	41
	Total	14.68	5.038	82
After	Control	13.54	4.278	41
	Dog	12.15	3.054	41
	Total	12.84	3.760	82

**Table 3:** Descriptive Statistics: Sadness.

	Group	Mean	Std. Deviation	N
Before	Control	11.12	4.854	41
	Dog	10.68	5.350	41
	Total	10.90	5.081	82
After	Control	10.34	4.264	41
	Dog	6.95	3.178	41
	Total	8.65	4.108	82

**Table 4:** Descriptive Statistics: General Positive Affect.

	Group	Mean	Std. Deviation	N
Before	Control	27.98	6.582	41
	Dog	26.71	6.772	41
	Total	27.34	6.667	82
After	Control	28.07	7.132	41
	Dog	30.37	7.031	41
	Total	29.22	7.132	82

**Table 5:** Descriptive Statistics: Joviality.

	Group	Mean	Std. Deviation	N
Before	Control	23.22	6.199	41
	Dog	23.15	6.097	41
	Total	23.18	6.110	82
After	Control	24.05	6.099	41
	Dog	29.29	5.951	41
	Total	26.67	6.543	82

**Table 6:** Descriptive Statistics: Anxiety and Gender.

	Condition	Gender	Mean	Std. Deviation	N
Before	Control	Female	42.11	14.19	19
		Male	40.91	12.22	22
		Total	41.46	13.02	41
	Dog	Female	37.94	12.97	21
		Male	41.67	13.31	20
		Total	39.76	13.11	41
	Total	Female	39.92	13.56	40
		Male	41.27	12.60	42
		Total	40.61	13.01	82
After	Control	Female	41.05	13.57	19
		Male	37.88	12.66	22
		Total	39.35	13.02	41
	Dog	Female	28.41	7.86	21
		Male	29.67	7.94	20
		Total	29.02	7.83	41
	Total	Female	34.42	12.55	40
		Male	33.97	11.34	42
		Total	34.19	11.87	82

**Table 7:** Descriptive Statistics: Negative Affect and Gender.

	Group	Gender	Mean	Std. Deviation	N
Before	Control	Female	15.52	5.297	21
		Male	12.90	3.959	20
		Total	14.24	4.821	41
	Dog	Female	14.26	3.754	19
		Male	15.86	6.289	22
		Total	15.12	5.269	41
	Total	Female	14.93	4.615	40
		Male	14.45	5.456	42
		Total	14.68	5.038	82
After	Control	Female	14.33	5.073	21
		Male	12.70	3.164	20
		Total	13.54	4.278	41
	Dog	Female	11.26	1.522	19
		Male	12.91	3.804	22
		Total	12.15	3.054	41
	Total	Female	12.88	4.084	40
		Male	12.81	3.473	42
		Total	12.84	3.760	82

**Table 8:** Descriptive Statistics: Sadness and Gender.

	Group	Gender	Mean	Std. Deviation	N
Before	Control	Female	11.14	4.778	21
		Male	11.10	5.057	20
		Total	11.12	4.854	41
	Dog	Female	9.79	4.404	19
		Male	11.45	6.045	22
		Total	10.68	5.350	41
	Total	Female	10.50	4.597	40
		Male	11.29	5.532	42
		Total	10.90	5.081	82
After	Control	Female	10.24	4.206	21
		Male	10.45	4.430	20
		Total	10.34	4.264	41
	Dog	Female	6.26	1.759	19
		Male	7.55	3.973	22
		Total	6.95	3.178	41
	Total	Female	8.35	3.813	40
		Male	8.93	4.397	42
		Total	8.65	4.108	82

**Table 9:** Descriptive Statistics: Positive Affect and Gender.

	Group	Gender	Mean	Std. Deviation	N
Before	Control	Female	27.52	7.692	21
		Male	28.45	5.336	20
		Total	27.98	6.582	41
	Dog	Female	28.42	6.818	19
		Male	25.23	6.524	22
		Total	26.71	6.772	41
	Total	Female	27.95	7.211	40
		Male	26.76	6.136	42
		Total	27.34	6.667	82
After	Control	Female	27.62	8.381	21
		Male	28.55	5.717	20
		Total	28.07	7.132	41
	Dog	Female	31.84	5.134	19
		Male	29.09	8.240	22
		Total	30.37	7.031	41
	Total	Female	29.63	7.263	40
		Male	28.83	7.071	42
		Total	29.22	7.132	82

**Table 10:** Descriptive Statistics: Joviality and Gender.

	Gender	Group	Mean	Std. Deviation	N
Before	Control	Male	23.70	5.536	20
		Female	22.76	6.877	21
		Total	23.22	6.199	41
	Dog	Male	21.68	6.267	22
		Female	24.84	5.580	19
		Total	23.15	6.097	41
	Total	Male	22.64	5.946	42
		Female	23.75	6.303	40
		Total	23.18	6.110	82
	Control	Male	24.45	5.790	20
After		Female	23.67	6.499	21
		Total	24.05	6.099	41
	Dog	Male	27.95	7.068	22
		Female	30.84	3.962	19
		Total	29.29	5.951	41
	Total	Male	26.29	6.653	42
		Female	27.08	6.486	40
		Total	26.67	6.543	82
